# Hiding in Plain Sight—Relapsing Polychondritis Disguised as Hip Arthritis: A Case Report and Literature Review

**DOI:** 10.1002/iid3.70159

**Published:** 2025-02-14

**Authors:** Zhanxia Li, Tao Ren

**Affiliations:** ^1^ Department of Respiratory and Critical Care Medicine Shanghai Sixth People's Hospital Affiliated to Shanghai Jiao Tong University School of Medicine Shanghai China

**Keywords:** airway stenosis, hip pain, relapsing polychondritis

## Abstract

**Background:**

Relapsing polychondritis (RP) is a rare autoimmune disorder characterized by episodic inflammation of cartilaginous tissues. Its diverse clinical manifestations frequently pose diagnostic challenges and lead to misdiagnosis.

**Methods:**

We present a 59‐year‐old female initially diagnosed with refractory hip osteoarthritis based on persistent left hip pain unresponsive to conventional anti‐inflammatory therapy. Subsequent development of stridor and dyspnea prompted comprehensive evaluation, including laryngotracheal imaging, autoimmune serology, and multidisciplinary consultation. Diagnostic criteria for RP were applied in accordance with revised Michet criteria.

**Results:**

Tracheal stenosis secondary to tracheal chondritis and iritis were identified, confirming the diagnosis of RP. Following initiation of corticosteroid therapy, the patient exhibited marked clinical improvement.

**Conclusion:**

This case underscores the importance of enhanced clinical awareness regarding atypical presentations of RP, particularly when accompanied by evolving systemic symptoms. A multidisciplinary diagnostic approach is advocated to facilitate early detection and improve patient outcomes.

## Graphical abstract

This article presents an unusual manifestation of relapsing polychondritis (RP), initially misdiagnosed as hip osteoarthritis. It underscores the importance of recognizing atypical presentations of RP and advocates for a multidisciplinary diagnostic approach to improve early detection and patient outcomes.

## 1 Introduction

Relapsing polychondritis (RP) is a rare, multisystem autoimmune disorder characterized by episodic inflammation of cartilage and proteoglycan‐rich tissues, such as the ears, nose, larynx, trachea, articular surfaces, heart, blood vessels, inner ear, and kidneys [1, 2]. When RP involves critical structures like the larynx and trachea, it can lead to severe respiratory complications and a poor prognosis. Due to its diverse and often subtle clinical presentations, RP is frequently misdiagnosed, resulting in delayed treatments that can adversely affect patient outcomes [3].

In particular, beginner and nonspecialist clinicians may overlook hallmark RP manifestations, which include auricular chondritis (erythematous or painful swelling of the external ear), nasal chondritis, respiratory tract involvement (laryngotracheal inflammation, airway stenosis), ocular inflammation (uveitis, scleritis), and seronegative polyarthritis [1, 4]. While knee and small joints (e.g., MCP, PIP) are commonly involved, large joint (including hip) involvement is less frequently reported but can occur [5]. According to the revised criteria by Michet and colleagues, a diagnosis of RP requires confirmation of inflammation in at least two of the following sites: auricular, nasal, or laryngotracheal cartilage. Alternatively, inflammation of one site plus two or more minor criteria—such as hearing loss, eye inflammation, or seronegative arthritis—can be used [6]. A recent publication underscores the importance of early and accurate recognition of these hallmark features in preventing misdiagnoses [7, 8].

Differentiating inflammatory arthritides like RP from degenerative conditions such as hip osteoarthritis (OA) can be challenging, especially in older patients. Hip pain in RP may present with synovitis and joint effusions rather than the classic joint space narrowing or osteophytes typical of primary OA. Comprehensive imaging (MRI, ultrasound, PET) and inflammatory markers often guide the distinction [9, 10].

This report highlights a patient initially misdiagnosed with unilateral hip OA. After months of persistent hip pain and newly emerging respiratory symptoms, she was eventually diagnosed with RP, illustrating the critical importance of a multidisciplinary, high‐index‐of‐suspicion approach for atypical presentations.

## 2 Case Presentation

### 2.1 Chief Complaint

A 59‐year‐old woman presented with a 4‐month history of left hip pain, cough, and sputum production, accompanied by intermittent dyspnea for the past 2 months.

### 2.2 Current Medical History

- Four months ago: The patient initially sought care for left hip pain and limited mobility. She was diagnosed with hip degeneration/arthritis but showed no improvement after anti‐infective and analgesic treatments.
- Two months ago: She developed a cough (3–4 episodes/week, lasting 2–3 h) and a foreign body sensation in her throat. No fever, chills, chest pain, or hemoptysis were noted. Lung CT and laryngoscopy at another hospital were unremarkable.
- One week before admission: The cough worsened, accompanied by chest tightness, dyspnea, and a small amount of white sputum.

### 2.3 Past Medical, Personal, and Family History

The patient had no significant past medical history. No notable personal or family history was reported.

### 2.4 Physical Examination at Admission

- Ophthalmic: Blurred vision and conjunctival congestion in the right eye.
- ENT: No saddle‐nose deformity; normal external ears and auricles without hearing loss.
- Respiratory: A small amount of inspiratory dry rales in both lungs.
- Musculoskeletal: Limited movement of the left hip joint with painful claudication.

### 2.5 Laboratory Tests

- Inflammatory markers:
  ○ Elevated erythrocyte sedimentation rate (ESR): 77 mm/h
  ○ Elevated IL‐6: 18.6 pg/mL (normal 0–5.3)
  ○ Elevated IL‐17A: 21.6 pg/mL (normal 0–20.6)
  ○ IL‐12: 4.9 pg/mL (normal 0–3.4)
  ○ Other markers in the interleukin family were normal. The assays were performed using an ELISA kit (Thermo Fisher Scientific, Waltham, MA, USA), as per manufacturer protocols.
- Other tests:
  ○ Comprehensive ANCA panel, antimyositis antibodies, immunoglobulins, rheumatoid factor: all normal.
  ○ Routine blood tests, C‐reactive protein (CRP), liver and kidney function, coagulation studies, interferon‐γ release assay, and arterial blood gas: all within normal limits.

### 2.6 Imaging and Other Diagnostic Tests

**Figure 1:**
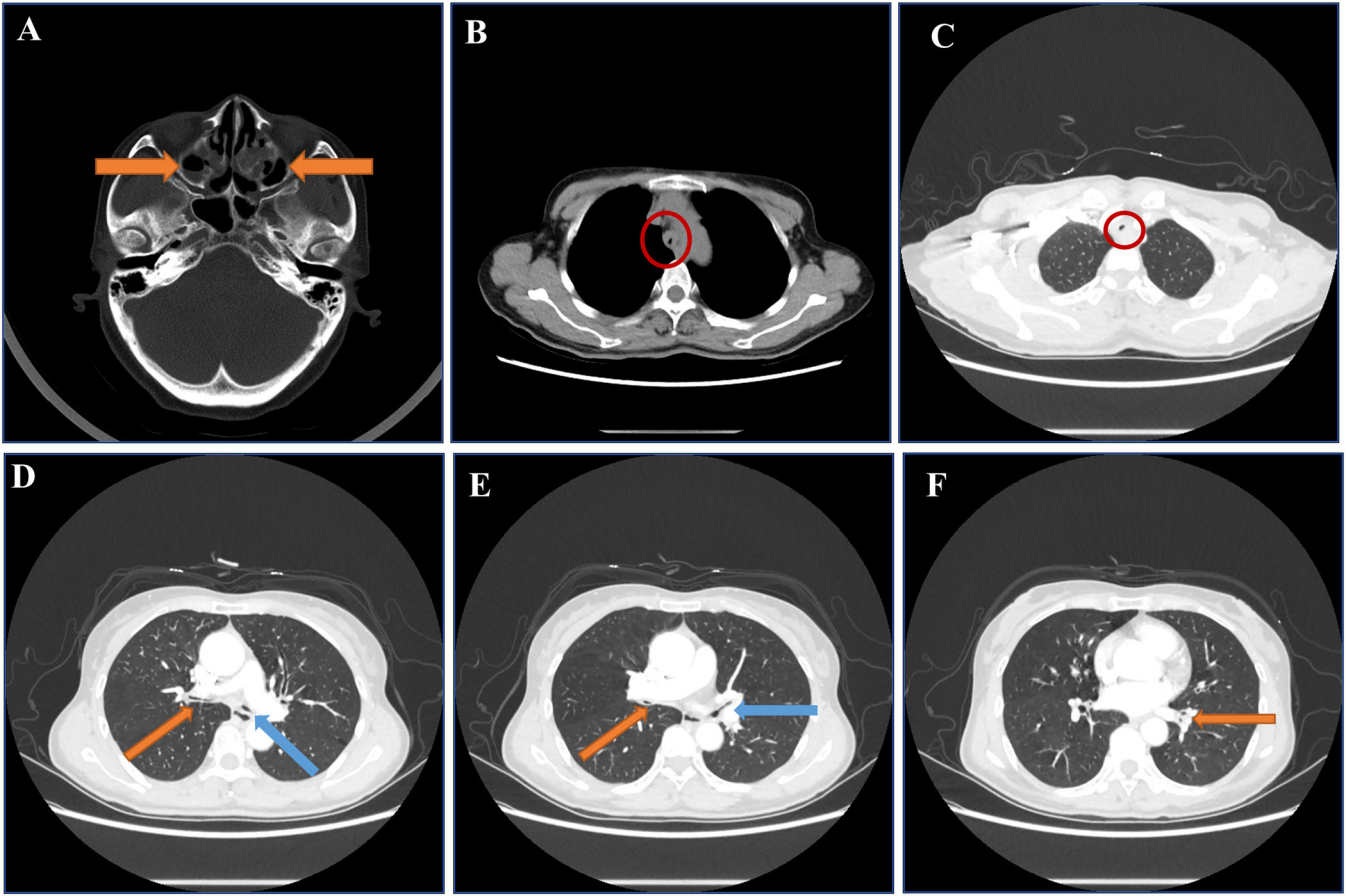
Sinus and chest CT scan. (A) Axial sinus CT scan demonstrated thickened mucous membranes in the bilateral maxillary and ethmoid sinuses (orange arrows), indicating sinus involvement. (B and C) Axial chest CT (Brilliance iCT; Philips Healthcare, Cleveland, Ohio, USA) scans revealed tracheal wall thickening (~5 mm) with tracheal lumen narrowing, with the narrowest portion measuring ~4–5 mm (red circles). (D) Axial chest CT scan indicated the narrowest points of the right (1–2 mm, orange arrow) and left main bronchi (1–2 mm, blue arrow). (E) Axial chest CT scan depicted narrowing of the right middle bronchus to about 1 mm (orange arrow) and the left upper lobe bronchus to ~2 mm (blue arrow). (F) Axial chest CT scan showed the left lower lobe bronchus narrowed to about 2 mm (orange arrow).

**Figure 2:**
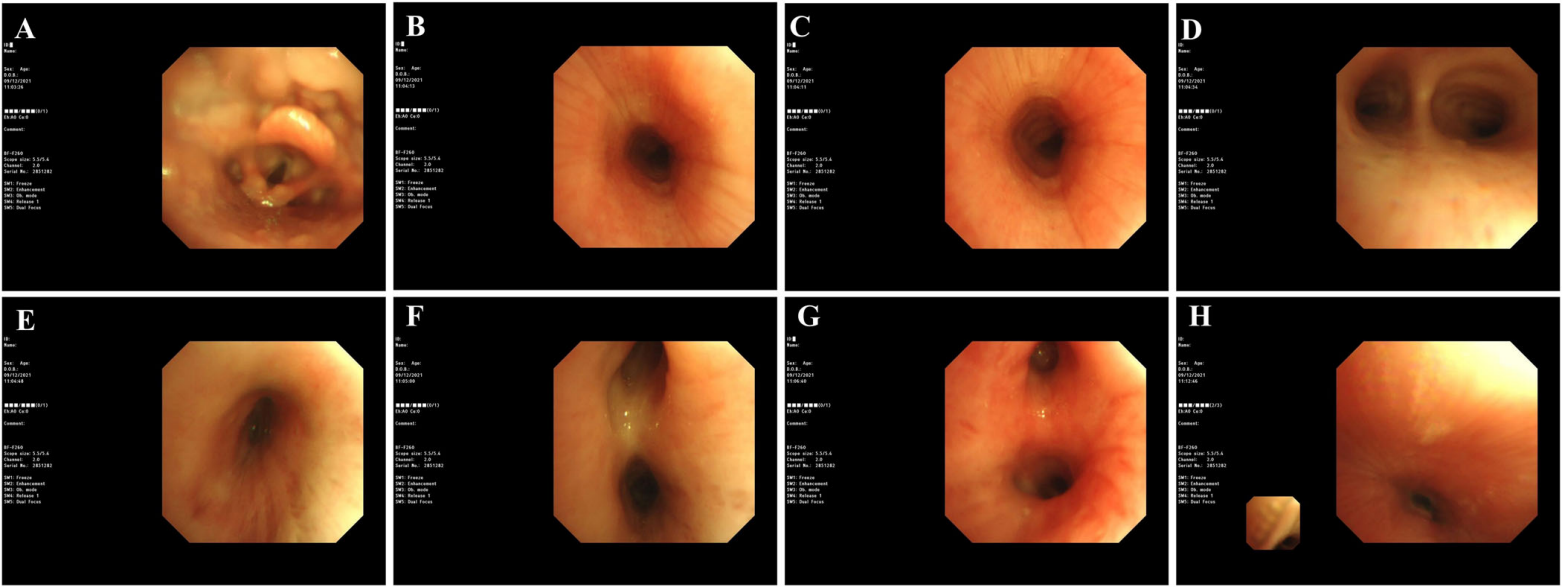
Bronchoscopic findings. (A) The glottis exhibited mild mucosal irregularities without significant obstruction. (B and C) The trachea showed concentric narrowing, with the cartilage rings absent, indicating severe airway involvement. (D) The carina of the trachea demonstrated both mucosal hypertrophy and atrophy, suggesting advanced inflammatory damage. (E) The right main bronchus revealed mucosal thickening and slight narrowing of the lumen. (F) The right middle bronchus showed marked mucosal thickening with luminal narrowing. (G) The left main bronchus presented with significant mucosal thickening and narrowing, similar to the right bronchus. (H) The trachea during the expiratory phase displayed generalized inflammation and mucosal edema, resulting in a narrowed airway passage.

**Figure 3:**
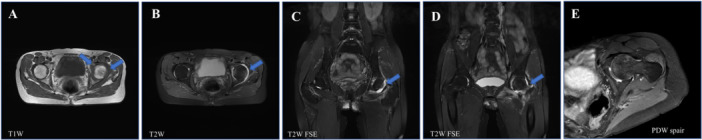
Pelvic magnetic resonance imaging. (A–D) Pretreatment MRI images showing left hip arthritis/synovitis, joint capsule, and surrounding soft tissue swelling. The images revealed: (A) Axial T1‐weighted image displayed joint effusion and abnormal signal in the glenoid labrum. (B) Axial T2‐weighted image showed increased fluid within the joint, indicating active inflammation. (C) Coronal T2‐weighted fat‐saturated image demonstrated narrowing of the joint space and poor continuity of the round ligament of the femoral head. (D) Coronal T2‐weighted fat‐saturated image highlighted patchy high signal intensity in the left femoral head and acetabulum, along with swelling of the surrounding soft tissues. (E) Follow‐up MRI, 3 months posttreatment, showed a reduction in joint cavity fluid and less pronounced joint space narrowing compared to previous imaging, indicating improvement in the inflammatory process.

- Sinus CT (Brilliance iCT; Philips Healthcare, Cleveland, Ohio, USA): Bilateral maxillary sinusitis, no nasal deformities (Figure 1A).
- Electronic nasopharyngoscopy (Olympus Medical Systems, Tokyo, Japan): Erosions and crusts in bilateral Little's area, hypertrophy of inferior turbinates, and chronic laryngeal congestion (Figure 2).
- Chest CT (Brilliance iCT; Philips Healthcare, Cleveland, Ohio, USA): Thickening of tracheal and bronchial walls with homogeneous enhancement; narrowing of the tracheal and bilateral main bronchial lumens. The narrowest tracheal lumen measured ~4 mm; the narrowest bronchiole lumen ~2 mm (Figure 1B–F).
- Bronchoscopy (BF‐F260; Olympus Medical Systems, Tokyo, Japan): Tracheal lumen narrowing, loss of cartilaginous rings, mucosal hypertrophy/atrophy, thickening of left and right lobe mucosa, widened left upper lobe and middle bronchial ridge (Figure 2). Biopsies showed chronic inflammation without malignancy.
- MRI (Signa Premier; GE Healthcare, Chicago, Illinois, USA) of the hip: Left hip OA/synovitis with joint capsule swelling and surrounding soft tissue involvement (Figure 3A–D).
- Ultrasonography (Mindray Resona; Mindray Medical International, Shenzhen, China): Thickened synovial membrane in the left hip joint cavity with fluid accumulation.
- Ophthalmology: Fundoscopy revealed right eye iritis.
- Cardiac Evaluation: ECG and echocardiogram: normal.
- Pulmonary Function (Jaeger PFT; Beijing Kingmed, Beijing, China): Mixed ventilation dysfunction, negative bronchodilator test; FVC 0.86 L (38.9% predicted), FEV1 25% predicted, FEV1/FVC 51.58%, and MVV 27.5% predicted.

### 2.7 Diagnosis and Treatment

Based on Damiani and Levine's criteria and the revised Michet criteria [6], the patient was diagnosed with RP. She was started on oral prednisone (30 mg/day) and cyclosporine (150 mg/day) with dosages tapered according to clinical response. The patient tolerated treatment well, with no significant adverse effects.

### 2.8 Follow‐Up Visits

- Three‐month follow‐up: The patient presented with a posttraumatic fracture after an accident. MRI showed improved left hip OA/synovitis (Figure 3E).
- Ten‐month follow‐up:
  ○ The patient's condition remained stable with no recurrence of symptoms. Cough and chest tightness had significantly improved, and blood tests showed normalized ESR and interleukin levels.
  ○ Pulmonary function:
    ■ FVC: 1.86 L (58.9% predicted)
    ■ FEV1: 65% predicted
    ■ FEV1/FVC: 71.58%
    ■ MVV: 57.5% predicted

These improvements negated the need for advanced respiratory interventions such as noninvasive ventilation or bronchoscopic stenting.

### 2.9 Ethical Considerations

All procedures involving human participants were conducted according to Institutional/National Research Committee Standards and the Helsinki Declaration (2013 revision). Written informed consent was obtained from the patient for the publication of this case report and accompanying images.

## 3 Discussion

RP was first documented in 1923 and formally named by Pearson and colleagues in 1960 [2, 11]. It has an estimated incidence of 3.5 cases per million per year [12]. Although RP can manifest at any age, it typically presents in adults between 40 and 60, with men and women equally affected [13]. Diagnostic delays are common, taking an average of 1.9–2.9 years in some regions [7]. The average time to diagnosis varied depending on the initial manifestations, with delays more pronounced in cases without classic auricular or nasal cartilage involvement [13].

The exact pathogenesis of RP remains unclear, but there is evidence that mechanical or inflammatory insults may expose cartilage matrix antigens, triggering immune‐mediated cartilage destruction. Genetic predispositions, such as HLA‐DR4 and HLA‐DR6, have been implicated, and infiltration of CD4+ T lymphocytes into cartilage supports an autoimmune basis [14, 15]. Elevated proinflammatory cytokines—including IL‐6, IL‐12, and IL‐17A—further underscore this immune‐mediated process [3, 16]. Notably, some patients may exhibit normal CRP levels despite active disease, highlighting the heterogeneity of acute‐phase responses in RP. Our patient's elevated ESR and cytokine levels align with active inflammation, despite a normal CRP that might reflect either disease variability or early‐stage disease activity.

In developing the present case report and discussion, we performed a focused literature review using PubMed and Embase databases. Our search encompassed articles published between [2010] and [2023], with the key terms “relapsing polychondritis,” “hip arthritis,” “airway involvement,” and “tracheal complications.” We also included older, seminal studies to ensure classical diagnostic criteria and historical insights were represented. Through this review, we identified the rarity of large joint (including hip) involvement, as well as the spectrum of airway lesions frequently encountered in RP—both of which guided our interpretation and discussion of this patient's atypical presentation.

RP commonly presents with auricular, nasal, and respiratory chondritis. However, joint involvement occurs in 50%–85% of cases, usually presenting as a nonerosive, nondeforming polyarthritis [7]. Hip involvement, though less common, should be considered when typical OA management fails, as illustrated by this case. Several case reports have documented RP mimicking OA in large joints, suggesting that inflammatory synovitis can be overlooked or misattributed to degenerative processes [17]. Furthermore, ocular complications occur in up to 60% of patients, ranging from mild conjunctivitis to severe uveitis [18, 19]. In particular, iritis or anterior uveitis, as seen in our patient, can be an early or isolated finding that further complicates diagnosis.

Respiratory involvement is a pivotal factor in RP, as airway lesions constitute the most common cause of death in this disease. Approximately 10% of patients initially present with respiratory symptoms—often leading to an average diagnostic delay of about 2.5 years—and up to 50% will experience respiratory involvement during the disease course [1]. Common manifestations include dyspnea, cough, stridor, and hoarseness. Laryngeal or tracheobronchial cartilage destruction can result in inspiratory distress, airway collapse, or even acute respiratory failure. On chest CT, hallmark features are airway wall thickening (> 2 mm), narrowing (≥ 25% lumen reduction), loss of cartilaginous rings (trachea bronchomalacia), and potential wall calcification or air trapping, often highlighted on expiratory‐phase scans [20]. Bronchoscopic evaluations frequently show widespread narrowing, missing cartilage rings, and mucosal changes (hypertrophy, edema, necrosis). In our patient, chest CT and bronchoscopy demonstrated these patterns, with histopathology confirming inflammatory damage.

Our case underscores how the absence of auricular/nasal chondritis initially obscured the RP diagnosis, as did the normal CRP levels. However, the subsequent finding of tracheal stenosis, loss of cartilaginous rings, and iritis was highly suggestive of RP. Notably, the improvement in tracheal narrowing observed on follow‐up imaging is somewhat atypical, given that airway damage in RP is classically described as progressive and potentially irreversible [20]. Nonetheless, early intervention with immunosuppressive therapy may have mitigated permanent structural damage, emphasizing the importance of high clinical suspicion and prompt treatment.

Granulomatosis with polyangiitis (GPA) represents an important differential diagnosis for patients presenting with upper respiratory tract and joint symptoms. Our patient had negative ANCA results, no granulomas on biopsy, and no characteristic sinus/nasal lesions. These factors effectively ruled out GPA. Notably, distinguishing between RP and ANCA‐associated vasculitides remains a critical step, as immunosuppressive regimens may differ in intensity and duration.

RP treatment aims to control inflammation, preserve organ function, and prevent irreversible damage. Glucocorticoids remain the mainstay for acute flares; immunosuppressants (cyclosporine, methotrexate, cyclophosphamide) or biologic agents (TNF inhibitors, tocilizumab, IL‐1 inhibitors, IL‐6 receptor antagonist) may be necessary for organ‐threatening or refractory disease [21, 22]. Evidence from recent observational cohorts suggested that early use of biologic therapies could improve respiratory outcomes, particularly in patients with severe airway involvement. In cases of significant airway compromise, local interventions (e.g., bronchoscopic stenting and balloon dilation) may be required.

Our patient improved significantly on a combination of prednisone and cyclosporine without needing advanced airway interventions. This underscores the potential for favorable outcomes when RP is recognized early and treated aggressively. However, longitudinal monitoring of both proinflammatory and anti‐inflammatory cytokines could enhance our understanding of disease relapses and therapy responses. Additionally, advanced imaging modalities such as contrast‐enhanced CT or PET–CT might better characterize subclinical inflammation and guide treatment decisions.

The main limitation of this case is the lack of serial cytokine measurements, which might have provided clearer insights into disease fluctuations and therapeutic response over time. While advanced imaging modalities such as ET–CT can offer additional information on subclinical inflammation, they are not always required if the clinical and conventional imaging findings are sufficient for diagnosis. Further research into reliable biomarkers for early detection and the comparative efficacy of newer biologic therapies—especially in atypical presentations like hip involvement—is warranted. Larger case series or prospective studies would help clarify the relationship between imaging findings, cytokine profiles, and long‐term clinical outcomes in RP.

## 4 Conclusion

RP is a rare yet potentially severe autoimmune disorder. Because of its protean manifestations, especially when presenting atypically (e.g., hip pain resembling OA), clinicians must maintain a high index of suspicion. This case demonstrates how prompt recognition, thorough differential diagnosis (including exclusion of GPA), and timely immunosuppressive therapy can halt disease progression and improve quality of life. By highlighting an unusual presentation with hip arthritis and partially reversible airway lesions, this report adds to the growing literature that underscores the heterogeneity of RP. Future studies should explore novel biomarkers for early detection and evaluate the long‐term efficacy of emerging therapeutic options, aiming to optimize care for patients with this challenging multisystem disease.

## Author Contributions

**Zhanxia Li:** data curation, formal analysis, investigation, methodology, visualization, writing – original draft. **Tao Ren:** conceptualization, funding acquisition, project administration, supervision, visualization, writing – review and editing.

## Data Availability

The authors have nothing to report.
